# Determination of ethanol in micro-volumes of blood by headspace gas chromatography: Statistical comparison between capillary and venous sampling sites

**DOI:** 10.1177/0025802420928632

**Published:** 2020-06-11

**Authors:** Luke Taylor, Vytautas Remeškevičius, Lili Saskoy, Tara Brodie, Jeshan Mahmud, Hannah Moir, James Brouner, Christopher Howe, Baljit Thatti, Sein O’ Connell, Gavin Trotter, Brian Rooney

**Affiliations:** 1 Applied and Human Sciences, Kingston University, UK; 2 Medical Bureau of Road Safety, University College Dublin, Ireland

**Keywords:** Toxicology, forensic science, blood alcohol, capillary blood, Road Traffic Act

## Abstract

Ethanol is the most commonly encountered drug in forensic toxicology, with widespread use throughout society. For this reason, it is important that there are a variety of reliable and robust methods to detect and quantify the content of alcohol in blood samples of suspected drink drivers. A common method of detection is gas chromatography with flame ionisation detector, with a number of sample preparation techniques employed. Typically, venous blood is sampled and used in the analysis. However, there is currently no legal specification in the UK of the blood sample source. This study investigates the use of capillary blood as an alternative to venous blood alongside two different sample volumes: 100 and 10 µL. Venous and capillary blood were collected from volunteers who had consumed alcohol. All blood sampling was carried out one hour after cessation of drinking. The results show a statistically significant difference between venous and capillary samples, with an average difference of 3.38 ± 1.99 mg/100 mL at 100 μL and approximately 4.13 ± 2.42 mg/100 mL at 10 μL, respectively. Predominantly, venous blood was detected at higher concentrations than the corresponding capillary samples. The deviations in alcohol samples between venous and capillary blood are consistent with previous studies. However, our research indicates that capillary blood is a viable matrix to test for alcohol, albeit one that underestimates blood-alcohol content in relation to venous sampling. There was no statistically significant difference between the 100 and 10 µL sample preparation methods on an individual basis, which infers that micro-volumes of alcohol are suitable for forensic blood-alcohol analysis.

## Introduction

Ethanol (also known as alcohol or ethyl alcohol) is a widely used recreational drug worldwide. A survey carried out in the UK in 2017 indicated that 57% of respondents admitted to using alcohol recreationally, which equates to approximately 29.2 million people nationwide.^1^ Ethanol acts as a depressant on the central nervous system and produces effects of relaxation, sedation, loss of inhibitions and impairment of motor coordination.^2^ Due to its effects and prominence in society, drink-driving limits were introduced to improve road safety and reduce road-traffic collisions.^3^ Alcohol limits in England and Wales were set at 80 mg/100 mL of blood, 35 µg/100 mL of breath and 107 mg/100 mL of urine.^4^ On 10 April 2015, the statutory option for drink driving was removed (section 8 subsection 2 of the Road Traffic Act 1988). Initially, this Act stated that if a breath specimen contained no more than 50 µg/100 mL ethanol, then the breath sample could be replaced with a sample of either blood or urine, and should an individual provide such a specimen, then the original breath specimen should be discarded.^3,5–7^ This option was originally brought in to compensate for issues with the reliability of the alcohol reading in breath samples. However, a review of the drink and drug driving laws by Sir Peter North in 2010 found that due to the increasing accuracy of evidential breath analysers, the statutory option was no longer necessary, and that an evidential breath reading alone is sufficient to ensure a conviction.^3^

Since the publication of the North report, the technique most commonly used to detect alcohol in road-traffic cases in the UK is the evidential breathalyser.^3^ Typically, a preliminary roadside test is carried out which, if failed, requires a further evidential breath test to be conducted under arrest at a police station. This involves the provision of two confirmatory breath specimens, the lowest of which is utilised.^5–7^ Despite the breath alcohol limit being 35 µg/100 mL, a prosecution limit of 40 µg/100 mL is routinely used.^3^ Moreover, the UK government is in the process of implementing mobile evidential breathalysers, which would mean gathering evidence at the roadside, without the need to go the police station to perform a final evidential breath test.^8,9^ However, frequently, there are issues with either the operation of the evidential breathalysers or the defendant’s ability to provide a breath sample. For example, in 2017, in the UK alone, 3862 people involved in road collisions refused or failed to give breath samples.^10^ In such cases, the police can charge the offender with failing to provide a sample or, more frequently, they can request a urine or blood sample. This is also the procedure that is followed if there is an issue with the operation of the evidential breathalyser. As a result, a significant number of road traffic cases still require the analysis of blood and urine in order to secure drink-drive convictions. In circumstances where blood samples are collected, this requires a forensic medical examiner or a trained health-care professional. The process of collecting venous blood for toxicological analysis may be invasive, time-consuming and difficult to achieve safely with an intoxicated, uncooperative suspect. Venous blood samples should be approximately 10 mL in volume and divided into two separate samples, one of which is offered to the suspect as their B sample.^11,12^ The collection of venous blood is carried out, as arterial blood-alcohol concentrations (BAC) are higher during the absorption phase compared to venous blood, while during the elimination phase, arterial blood alcohol is lower than venous blood alcohol. Furthermore, the puncturing of arteries is not recommended.^13^

A potential alternative sample matrix is capillary blood. This is a less invasive method of sampling blood, taken commonly from a puncture on the finger. Capillary blood is a mixture of venous and arterial blood.^14^ However, is not presently utilised for analysis of ethanol in road-traffic toxicology, as the relationship between alcohol levels in capillary blood compared to venous blood is not well defined, with only a limited number of studies conducted to date.^15,16^ The most commonly utilised laboratory technique for the detection of alcohol is gas chromatography with flame ionisation detector (GC-FID).^17–19^ This technique is rapid and reliable and does not require any significant sample preparation or extraction.^20^

With the increasing sensitivity of analysis due to advances in instrumentation, smaller volumes of samples can be used. This includes micro-sampling and alternative biological samples which are the subject of ongoing research in forensic toxicology.^21,22^ The benefits of these methods could include a less invasive and faster sample-collection process along with a requirement for smaller sample volumes. Micro-samples for analysis of blood alcohol have previously been demonstrated using volumes as low as 20–50 μL with proton nuclear magnetic resonance (^1^H-NMR)^23^ and GC-FID.^24,25^ However, frequently, casework laboratories tend to use larger volumes for GC-FID due to issues with intra-sample uncertainty, with typical samples volumes of up to 0.1–1 mL analysed.

The aims of this study were to examine the relationship between capillary and venous blood alcohol and to investigate whether capillary blood could act as an alternative to venous blood sampling. The use of capillary blood samples could lead to a reduction in sampling times and a simpler and more efficient sample-collection process. Due to the relatively small volume of capillary blood samples, an effective analysis will require a micro-sampling technique to analyse as little as 10 µL of blood.

## Methods

### Reagents and materials

Aqueous ethanol standard solutions at concentrations of 10, 20, 50, 100, 200 and 400 mg/100 mL from Cerilliant (Round Rock, TX) were used. Aqueous ethanol Certified Reference Material quality control (QC) solutions at concentrations of 20, 80 and 200 mg/100 mL from LGC European Reference Materials (London, UK) acted as QC samples. Anhydrous tertiary butanol and sodium metabisulphite from Thermo Fisher Scientific (Waltham, MA) were used as internal standard and antioxidant, respectively. The vials used for collecting and storing blood were 5 mL Labco vials (Lampeter, UK) with sodium fluoride/potassium oxalate for venous blood, and 300 μL SARSTEDT Microvette CB 300 K2E tubes (Newton, NC) with EDTA dipotassium salt for capillary blood. Internal standard was made by using 500 mL distilled water, adding 25 μL of tertiary butanol and 2.5 g of sodium metabisulphite.

### Study design

The study protocol was approved by the Faculty Research Ethics Committee (FREC) of Kingston University London (ethics code: 1819063.1). All participants provided informed consent and signed consent forms to take part in the study. The volunteers were healthy individuals aged 20–45 years accustomed to social and moderate drinking, ranging in height from 165 to 185 cm and in weight from 60 to 100 kg. Prior to commencing the experiment, all participants were not monitored. No instructions were given on what and when they could eat or drink (with the exception of alcohol) before the start of the study.

During the study, the participants were given the choice of two different alcoholic beverages: a beer at 4.8% alcohol by volume (ABV) or a pre-mixed gin and tonic at 5% ABV. Male volunteers chose to consume the beer, whilst the female volunteers selected the gin and tonic mix. The male participants were provided with a volume of 568 or 1136 mL of beer, while female participants were provided with a volume of 250 or 500 mL of the gin and tonic mix. Participants completed drinking within a 40-minute period. The blood samples from participants were collected one hour after cessation of drinking. Throughout the study until completion of sample collections, participants were instructed not to drink, eat, urinate or smoke. Samples 1–3, 5, 7, 22–31, 34, 38 and 39 were from males who drank two 4.8% ABV pint measures (568 mL) of beer, while samples 6, 10–19, 21, 33, 35, 36 and 40 were from male participants who drank one 4.8% ABV pint measure (568 mL) of beer. Samples 8, 9, 20, 32 and 37 were from female participants who drank two 250 mL measures of 5% ABV gin and tonic, while sample 4 was from a female participant who drank one 250 mL measure of 5% ABV gin and tonic.

Before taking the blood sample, the sampling area was disinfected with wipes containing isopropanol. Approximately 5 mL of whole blood was taken from an antecubital vein in a seated position using a disposable BD Vacutainer® Safety-Lok blood collection set (Franklin Lakes, NJ) with an attached vial holder and collected into 5 mL Labco vials with sodium fluoride and potassium oxalate preservative. These vials contain a minimum of 1% sodium fluoride and potassium oxalate. The vials and preservatives used in this study are the same make and manufacturer as those contained with the road-traffic sample collection kit used by police forces in England. Approximately 3–5 mL of blood was collected, with a total vial capacity of 5 mL. Capillary samples were obtained by using a disposable lancet to draw blood. This was taken from the index finger. This area was disinfected using isopropanol wipes prior to sampling, and the lanced site was palpated to aid blood flow during the sample-collection process. Two CB300K2E tubes, amounting to approximately 600 µL, were taken per participant. This volume was required to ensure there was sufficient sample for duplicate analysis in 100 and 10 µL batches. Once sample collection was complete, the samples were analysed by GC-FID within 24 hours of collection. Capillary blood was transferred from the original Microvette containers into sealed 1.5 mL glass vials using glass Pasteur pipettes. The samples were then pipetted into headspace vials using Gilson Microman M100 or M10 positive displacement pipettes and tips (Middleton, WI). Excess samples were used for repeat analysis in circumstances where QCs fell outside the acceptance range. Once sample analysis was complete, samples were destroyed as per Human Tissue Authority guidelines.

For the first half of the samples (samples 1–18), venous blood was taken first followed by capillary blood, while for the remaining samples (samples 19–40), capillary blood was taken first followed by venous blood. This was done to determine if the delay associated with venous sampling before capillary blood would affect the difference between venous and capillary blood alcohol. Venous and capillary blood samples were taken from the same arm during the blood draw, with capillary blood being taken from the index finger as well as the ring finger if the required amount of blood was not collected from one finger.

Thirteen volunteers (eight male and five female) were used in this study, with a number of volunteers providing more than one sample on separate sampling days. Of the male volunteers, seven were Caucasian and one was of Asian descent. The Caucasian volunteers were primarily from the UK, the Republic of Ireland and Eastern Europe; the Asian volunteer was of Bangladeshi origin. Of the female volunteers, one volunteer was of Middle Eastern origin, one was of Asian origin and three were Caucasian. The Caucasian volunteers were of Iranian, Irish, Sri Lankan and Italian descent.

### Sample preparation

Calibrants, samples and QCs were made by pipetting 1 mL of internal standard using an Eppendorf Research Pro 50 μL–1 mL electronic pipette (Hamburg, Germany) to 20 mL headspace vials and spiking it with 100 μL of sample or calibrator or QC using a Gilson Microman M100 positive displacement pipette. Aqueous QC samples were run after calibration end and at the end of each batch with concentrations of 20, 80 and 200 mg/100 mL. For micro-sampling, 100 μL of internal standard using the same automatic pipette as the 100 μL batches was used and spiked with 10 μL of either sample or calibrant or QC using a Gilson Microman M10 positive displacement pipette. All samples were run in duplicate using split flow with two columns and two detectors. Four quantitative values per sample were obtained.

### Instrumentation

A Shimadzu GC-2014 (Kyoto, Japan) with RTX BAC 1 (30 m with 0.32 mm ID) and RTX BAC 2 (30 m with 0.32 mm ID) dual column with a HTA 200 Headspace Autosampler (Brescia, Italy) were used. Helium carrier gas, a hydrogen FID fuel source, blank air to maintain FID flame ignition and nitrogen make-up gas were used. The GC-FID and headspace parameters are shown in Table 1.

**Table 1.** Gas chromatography and headspace sampler parameters for the analysis of ethanol in blood.

Parameter	Value	
Inlet temperature	110°C	
Injection mode	Split	
Pressure	85 kPa	
Column flow	2.78 mL/min	
Linear velocity	42.30 cm/s	
Purge flow	3.00 mL/min	
Split ratio	5.00	
Oven temperature	40°C isothermal	
Oven temperature (headspace sampler)	60°C	
Syringe temperature	70°C	
Fill volume	1.75 mL	
Oscillation time	0.50 minutes on	0.10 minutes off
Sample speed	5.0 mL/min	
Injection speed	80 mL/min	
Sample speed	5.0 mL/min	

The method was designed specifically for quantitation of ethanol in blood samples. This method was validated prior to the study initialisation.

### Data analysis and statistical analysis

The data analysis for the calibration curves, QCs and sample concentrations was carried out using Shimadzu GC solutions software. Microsoft Excel was used to carry out statistical analysis using averages, standard deviations (*SD*s), *p*-values and *t*-tests. A paired two-tailed *t*-test was employed to check the significance of differences between the mean values, with values ≤0.05 indicating a significant difference between means. Values were compared on an individual basis comparing the duplicate values of each sample (a total of four measurements and three degrees of freedom). The entire sample population data were analysed using SPSS software version 26. The population data were tested with the Kolmogorov–Smirnov test for normality. For sample subsets that were not normally distributed, a non-parametric test Wilcoxon matched-pair signed rank test was used to analyse the significance of difference, with a *p*-value of <0.05 indicating a significant difference. Coefficient of variance (CV) was used as a measure of variability, as a high CV typically equates to a high variation of duplicate values in relation to the *SD* and the mean. *SD* was calculated using the function:

$$\sigma =\sqrt{\frac{\sum {\left(X-\mu \right)}^{2}}{n}}$$

where σ is the population *SD*, ∑ is the sum, *μ* is the population mean and *n* is the number of values within the data set. CV was calculated using:

$$CV=\frac{SD}{\mathrm{Mean}}\times \;100\%$$

## Results

### Investigation of the effects of reduced sample volume on the quantitation of blood alcohol in venous and capillary samples

Analysis of BAC was carried out on samples of 100 μL of venous blood and capillary blood. The analysis was then repeated on the same samples, with the sample volume reduced to 10 μL. All calibration curves had a *R*^2^ value of >0.999, and all QCs were within 3% of the certificate of analysis value. All CVs and *SD*s were <3% for all QCs, with the exception of QC 20 mg/100 mL, where *SD* alone was a more appropriate measurement. Our results indicate that a 10-fold reduction in volume from 100 to 10 μL produces no statistically significant difference in the measured alcohol value in either venous or capillary blood samples on an individual sample-by-sample basis. However, a statistically significant difference was found for the differing sample volumes of capillary blood when comparing the entire sample population. Despite this, on an individual sample-by-sample comparison, the BACs of 74% of samples were not significantly different.

The statistically significant difference in the overall sample subset for 100 μL compared to 10 μL capillary volumes is due to the consistent trend in which the 100 μL samples have a higher measured BAC. However, the average mean difference between the two sampling volumes was found to be just 0.41 mg/100 mL in venous and 1.21 mg/100 mL in capillary blood samples. The CV and *SD* values were similar for the different sampling volumes (Tables 2 and 3), with the average CV for all 10 μL volume samples being 2.87%, while for all 100 μL volume samples, it was 3.76%. This suggests that sample volumes as low as 10 μL are still able to quantify ethanol content accurately in both venous and capillary blood, and indicates that the methods utilising reduced sample volume can perform at the same standard as the traditional higher-sample volume methods.

**Table 2.** Comparison of blood-ethanol concentrations determined using 10 or 100 µL aliquots of venous whole blood.

Sample	Mean BAC for 100 μL volume (mg/100 mL)	*SD*	CV%	Mean BAC for 10 μL volume (mg/100 mL)	*SD*	CV%	Difference (mg/100 mL)	Paired *t*-test *p*-value
1 (M)	65	1.45	2.23	64	2.29	3.56	0.73	0.649
2 (M)	74	1.33	1.79	75	1.07	1.43	0.29	0.820
3 (M)	56	0.99	1.77	56	2.20	3.90	0.69	0.645
4 (F)	7^a^	n/a	n/a	n/a	n/a	n/a	n/a	n/a
5 (M)	57	0.37	0.65	58	0.72	1.24	0.96	0.194
6 (M)	20	0.45	2.23	20	0.23	1.12	0.35	0.128
7 (M)	67	0.78	1.17	71	0.32	0.45	3.52	0.005
8 (F)	26	0.60	2.33	27	1.26	4.65	1.29	0.245
9 (F)	26	0.54	2.07	27	0.45	1.67	0.93	0.009
10 (M)	23	0.99	4.29	21	0.52	2.45	1.89	0.037
11 (M)	19	1.28	6.63	19	0.92	4.86	0.42	0.254
12 (M)	17	1.44	8.61	16	0.72	4.64	1.18	0.101
13 (M)	28	0.51	1.83	28	0.79	2.87	0.09	0.742
14 (M)	31	1.12	3.60	30	1.22	4.05	0.88	0.399
15 (M)	19	2.17	11.48	19	0.55	2.91	0.01	0.606
16 (M)	27	1.29	4.81	27	0.73	2.68	0.44	0.629
17 (M)	25	0.81	3.24	26	1.07	4.17	0.52	0.335
18 (M)	29	1.16	4.01	29	1.17	4.03	0.01	0.984
19 (M)	25	0.97	3.81	23	0.97	4.25	2.49	0.038
20 (F)	31	1.42	4.62	30	0.07	0.25	1.10	0.485
21 (M)	25	1.55	6.19	27	1.04	3.87	1.75	0.299
22 (M)	64	0.37	0.58	62	0.74	1.19	2.31	0.392
23 (M)	58	1.11	1.90	52	1.11	2.13	6.08	0.007
24 (M)	67	1.02	1.52	62	0.70	1.13	5.21	0.087
25 (M)	59	1.22	2.07	58	1.01	1.74	0.61	0.435
26 (M)	76	0.70	0.92	74	1.41	1.89	1.47	0.195
27 (M)	72	0.84	1.17	73	1.88	2.60	0.41	0.531
28 (M)	42	1.88	4.44	43	0.68	1.57	0.91	0.563
29 (M)	64	2.33	3.66	62	1.13	1.81	1.51	0.496
30 (M)	64	1.41	2.20	67	2.81	4.18	2.75	0.224
31 (M)	63	2.26	3.57	65	1.16	1.80	1.35	0.365
32 (F)	32	1.92	6.07	33	0.79	2.36	1.76	0.133
33 (M)	21	1.01	4.74	20	0.59	2.88	1.09	0.322
34 (M)	73	0.46	0.63	72	1.01	1.41	0.74	0.220
35 (M)	31	0.27	0.84	30	0.09	0.30	1.56	0.011
36 (M)	21	0.55	2.60	18	1.15	6.32	2.85	0.077
37 (F)	35	0.22	0.63	35	0.34	0.97	0.56	0.350
38 (M)	65	0.88	1.34	66	0.68	1.03	1.28	0.258
39 (M)	63	1.22	1.93	62	1.14	1.83	0.73	0.659
40 (M)	36	1.06	2.97	33	0.96	2.92	2.77	0.009

Our results indicate that a 10-fold dilution in sample volume does not result in significant difference in detected alcohol concentration of this sample matrix.

^a^Value was below the limit of quantitation of the method.

BAC: blood-alcohol concentration; *SD*: standard deviation; CV: coefficient of variance; M: male; F: female.

**Table 3.** Comparison of blood-ethanol concentration determined using 10 or 100 µL aliquots of capillary (fingertip) blood.

Sample	Mean BAC for 100 μL volume (mg/100 mL)	*SD*	CV%	Mean BAC for 10 μL volume (mg/100 mL)	*SD*	CV%	Difference (mg/100 mL)	Paired *t*-test *p*-value
1 (M)	61	1.24	2.03	60	1.62	2.69	0.76	0.579
2 (M)	69	1.29	1.88	67	1.67	2.51	2.15	0.028
3 (M)	51	1.90	3.76	48	0.54	1.12	2.45	0.119
4 (F)	6^a^	n/a	n/a	n/a	n/a	n/a	n/a	n/a
5 (M)	52	0.83	1.58	55	1.23	2.25	2.44	0.072
6 (M)	14	0.58	4.11	15	0.34	2.23	1.21	0.039
7 (M)	60	1.06	1.79	60	0.65	1.08	0.68	0.404
8 (F)	20	0.59	2.93	21	0.48	2.31	0.68	0.148
9 (F)	21	0.35	1.71	21	0.27	1.30	0.39	0.070
10 (M)	19	0.73	3.87	16	0.65	4.00	2.64	0.000
11 (M)	14	0.91	6.43	12	0.72	6.30	2.63	0.098
12 (M)	11	1.05	9.19	9*	0.84	9.01	2.02	0.005
13 (M)	25	1.26	4.95	26	1.88	7.33	0.14	0.942
14 (M)	29	1.46	5.10	27	1.24	4.59	1.68	0.140
15 (M)	20	0.89	4.48	19	0.50	2.68	1.25	0.094
16 (M)	25	1.35	5.36	24	0.96	4.03	1.34	0.031
17 (M)	23	1.06	4.57	23	0.88	3.90	0.60	0.150
18 (M)	26	0.78	2.99	24	1.58	6.57	2.12	0.378
19 (M)	21	0.13	063	21	1.34	6.46	0.03	0.197
20 (F)	29	0.35	1.19	27	1.09	4.10	2.68	0.086
21 (M)	22	0.65	2.98	21	1.24	5.84	0.56	0.726
22 (M)	61	0.07	0.11	57	0.99	1.75	4.66	0.024
23 (M)	54	0.00	0.02	51	1.83	3.60	3.32	0.153
24 (M)	62	0.56	0.90	60	0.54	0.90	2.31	0.034
25 (M)	54	0.71	1.33	52	0.85	1.63	1.37	0.134
26 (M)	71	0.89	1.25	69	2.11	3.08	2.14	0.157
27 (M)	73	0.67	0.91	71	0.91	1.29	2.05	0.077
28 (M)	39	2.34	5.95	41	1.58	3.83	1.80	0.704
29 (M)	58	1.71	2.98	58	0.48	0.84	0.34	0.240
30 (M)	61	0.70	1.15	64	1.02	1.59	2.97	0.010
31 (M)	61	3.56	5.83	59	1.49	2.50	1.82	0.288
32 (F)	29	1.11	3.83	30	2.03	6.84	0.66	0.584
33 (M)	21	0.49	2.38	19	0.52	2.74	1.89	0.127
34 (M)	68	0.94	1.38	67	0.33	0.48	0.91	0.255
35 (M)	30	0.77	2.53	29	0.17	0.60	1.93	0.122
36 (M)	20	0.22	1.09	18	0.75	4.26	2.20	0.012
37 (F)	33	0.36	1.11	28	1.07	3.78	4.29	0.028
38 (M)	65	0.99	1.53	60	0.84	1.39	4.28	0.066
39 (M)	60	1.06	1.75	57	1.58	2.75	2.94	0.283
40 (M)	32	0.83	2.57	30	0.26	0.89	2.58	0.048

Capillary whole-blood samples were analysed at two different volumes. Our results indicate that dilution of the sample volume from 100 to 10 µL does not result in a significant difference in reported alcohol value of this sample matrix.

^a^Value was below the limit of quantitation of the method.

### Comparison of venous and capillary blood using standard sample volumes and reduced sample volumes

The differences in ethanol concentration for venous blood sampling and capillary blood sampling were investigated using sample volumes of 100 and 10 μL (Tables 4 and 5). Our results indicated that there was a statistically significant difference in alcohol concentration of capillary blood samples compared to venous blood samples, regardless of what sample volume was analysed. Aliquots of 100 μL of venous and 100 μL of capillary blood from the same donor sampled at the same time was analysed for alcohol. The average concentration difference was found to be 3.38 mg/100 mL – higher in venous blood. For the 10 μL sample aliquots, the average BAC was 4.13 mg/100 mL higher in venous blood. The range of variation for the 100 μL aliquots was 0.58–7.44 mg/100 mL. For the 10 μL sample volumes, the range of variation was 0.23–10.94 mg/100 mL. All venous samples had higher ethanol concentrations than their corresponding capillary samples, with the exception of samples 15 and 27 (Table 4). In these samples, capillary ethanol was greater by 1.01 and 0.93 mg/100 mL, respectively. However, both of these increased concentrations are within normal analytical variation limits.

**Table 4.** Comparison of ethanol concentrations in samples of venous and capillary blood using 100 µL aliquots.

Sample	Mean BAC for 100 μL venous (mg/100 mL)	*SD*	CV%	Mean BAC for 100 μL capillary (mg/100 mL)	*SD*	CV%	Difference (mg/100 mL)	Paired *t*-test *p*-value
1 (M)	65	1.45	2.23	61	1.24	2.03	4.01	0.028
2 (M)	74	1.33	1.79	69	1.29	1.88	5.61	0.014
3 (M)	56	0.99	1.77	51	1.90	3.76	5.09	0.028
4 (F)	7^a^	n/a	n/a	6^a^	n/a	n/a	n/a	n/a
5 (M)	57	0.37	0.65	52	0.83	1.58	4.55	0.007
6 (M)	20	0.45	2.23	14	0.58	4.11	5.95	0.000
7 (M)	67	0.78	1.17	60	1.06	1.79	7.44	0.001
8 (F)	26	0.60	2.33	20	0.59	2.93	5.70	0.000
9 (F)	26	0.54	2.07	21	0.35	1.71	5.48	0.000
10 (M)	23	0.99	4.29	19	0.73	3.87	4.28	0.002
11 (M)	19	1.28	6.63	14	0.91	6.43	5.14	0.003
12 (M)	17	1.44	8.61	11	1.05	9.19	5.29	0.000
13 (M)	28	0.51	1.83	25	1.26	4.95	2.16	0.094
14 (M)	31	1.12	3.60	29	1.46	5.10	2.46	0.034
15 (M)	19	2.17	11.48	20	0.09	4.48	–1.01	0.552
16 (M)	27	1.29	4.81	25	1.35	5.36	1.65	0.000
17 (M)	25	0.81	3.24	23	1.06	4.57	1.85	0.036
18 (M)	29	1.16	4.01	26	0.78	2.99	2.83	0.075
19 (M)	25	0.97	3.81	21	0.13	0.63	4.64	0.114
20 (F)	31	1.42	4.62	29	0.35	1.19	1.44	0.251
21 (M)	25	1.55	6.19	22	0.65	2.98	3.34	0.037
22 (M)	64	0.37	0.58	61	0.07	0.11	2.83	0.012
23 (M)	58	1.11	1.90	54	0.00	0.02	4.30	0.012
24 (M)	67	1.02	1.52	62	0.56	0.90	5.07	0.096
25 (M)	59	1.22	2.07	54	0.71	1.33	5.15	0.019
26 (M)	76	0.70	0.92	71	0.89	1.25	5.06	0.008
27 (M)	72	0.84	1.17	73	0.67	0.91	–0.93	0.332
28 (M)	42	1.88	4.44	39	2.34	5.95	3.04	0.082
29 (M)	64	2.33	3.66	58	1.71	2.98	6.10	0.006
30 (M)	64	1.41	2.20	61	0.70	1.15	3.17	0.014
31 (M)	63	2.26	3.58	61	3.56	5.83	2.01	0.524
32 (F)	32	1.92	6.07	29	1.11	3.83	2.53	0.010
33 (M)	21	1.01	4.74	21	0.49	2.38	0.69	0.361
34 (M)	73	0.46	0.63	68	0.94	1.38	4.74	0.002
35 (M)	31	0.27	0.84	30	0.77	2.53	0.96	0.204
36 (M)	21	0.55	2.60	20	0.22	1.09	1.20	0.013
37 (F)	35	0.22	0.63	33	0.36	1.11	2.73	0.102
38 (M)	65	0.88	1.34	65	0.99	1.53	0.58	0.766
39 (M)	63	1.22	1.93	60	1.06	1.75	2.80	0.128
40 (M)	36	1.06	2.97	32	0.83	2.58	3.42	0.015

Our results indicate that capillary and venous blood provide differing BAC values and cannot be treated as equivalent matrices. In approximately 92.5% of samples, the corresponding capillary BAC was on average 3.38 mg/100 mL lower than the venous equivalent.

^a^Value was below the limit of quantitation of the method.

**Table 5.** Comparison of ethanol concentrations in samples of venous and capillary blood using 10 µL aliquots.

Sample	Mean BAC for 10 μL venous (mg/100 mL)	*SD*	CV%	Mean BAC for 10 μL capillary (mg/100 mL)	*SD*	CV%	Difference (mg/100 mL)	Paired *t*-test *p*-value
1 (M)	64	2.29	3.56	60	1.62	2.69	4.05	0.168
2 (M)	75	1.07	1.43	67	1.67	2.51	8.05	0.015
3 (M)	56	2.20	3.9	48	0.54	1.12	8.23	0.007
4 (F)	n/a^a^	n/a	n/a	n/a	n/a	n/a	n/a	n/a
5 (M)	58	0.72	1.24	55	1.23	2.25	3.07	0.044
6 (M)	20	0.23	1.12	15	0.34	2.23	5.08	0.000
7 (M)	55	0.68	1.24	44	0.58	1.33	10.94	0.000
8 (F)	27	1.26	4.65	21	0.48	2.31	6.31	0.002
9 (F)	27	0.45	1.67	21	0.27	1.30	6.02	0.000
10 (M)	21	0.52	2.45	16	0.65	4.00	5.03	0.000
11 (M)	19	0.92	4.86	12	0.72	6.30	7.35	0.008
12 (M)	16	0.72	4.64	9*	0.84	9.01	6.14	0.000
13 (M)	28	0.79	2.87	26	1.88	7.33	1.93	0.061
14 (M)	30	1.22	4.05	27	1.24	4.59	3.27	0.000
15 (M)	19	0.55	2.91	19	0.50	2.69	0.23	0.647
16 (M)	27	0.73	2.68	24	0.96	4.03	3.43	0.008
17 (M)	26	1.07	4.17	23	0.88	3.90	2.97	0.037
18 (M)	29	1.17	4.03	24	1.58	6.57	4.94	0.047
19 (M)	23	0.97	4.25	21	1.34	6.46	2.11	0.094
20 (F)	30	0.07	0.25	27	1.09	4.10	3.02	0.052
21 (M)	27	1.04	3.87	21	1.24	5.84	5.65	0.013
22 (M)	62	0.74	1.19	57	0.99	1.75	5.18	0.014
23 (M)	52	1.11	2.13	51	1.83	3.60	1.55	0.382
24 (M)	62	0.70	1.13	60	0.54	0.90	2.17	0.039
25 (M)	58	1.01	1.74	52	0.85	1.63	5.91	0.008
26 (M)	74	1.41	1.89	69	2.11	3.08	5.74	0.006
27 (M)	73	1.88	2.60	71	0.91	1.29	1.53	0.407
28 (M)	43	0.68	1.57	41	1.58	3.83	2.16	0.106
29 (M)	62	1.13	1.81	58	0.48	0.84	4.24	0.043
30 (M)	67	2.81	4.18	64	1.02	1.59	2.96	0.148
31 (M)	65	1.16	1.80	59	1.49	2.50	5.18	0.024
32 (F)	33	0.79	2.36	30	2.03	6.84	3.63	0.038
33 (M)	20	0.59	2.88	19	0.52	2.74	1.48	0.088
34 (M)	72	1.02	1.41	67	0.33	0.48	4.91	0.032
35 (M)	30	0.09	0.30	29	0.17	0.60	1.33	0.129
36 (M)	18	1.15	6.33	18	0.75	4.26	0.55	0.513
37 (F)	35	0.34	0.97	28	1.06	3.78	6.46	0.089
38 (M)	66	0.68	1.03	60	0.84	1.39	6.14	0.095
39 (M)	62	1.14	1.83	57	1.58	2.75	5.00	0.243
40 (M)	33	0.96	2.92	30	0.26	0.89	3.23	0.032

Our results indicate that micro-analyses of capillary and venous blood provide differing BAC values and cannot be treated as equivalent matrices. In approximately 97.5% of samples, the corresponding capillary BAC was on average 4.13 mg/100 mL lower than the venous equivalent.

^a^Value was below the limit of quantitation of the method.

## Discussion

Our results indicate that the use of micro-sampling and reduced sample volume does not affect the accuracy of alcohol quantitation in blood samples when tested by GC-FID. It should also be noted that alcohol in capillary blood samples was quantified on average 3.76 mg/100 mL lower than corresponding venous samples. This raises the possibility of capillary blood samples and a corresponding micro-sample analysis method being used in the course of road-traffic toxicology casework, where rapid sampling and high-accuracy quantitative analysis is required. Although it is clear that capillary samples may present an underestimation of the motorist’s BAC, this is offset by the more rapid collection procedure. The typical elimination rates of alcohol are between 15 and 25 mg/100 mL/h. Therefore, any significant sampling delay can lead to an underestimation of the suspect’s BAC at the time of the incident.^26^ In addition to this, one of the major obstacles to obtaining a blood sample is a suspect stating a fear (real or contrived) of needles and/or a failure of the health-care professional to extract the required sample blood successfully. While urine sampling is an alternative, the procedure is time-consuming and requires more manpower from law enforcement, as the suspect has to urinate first and then provide an evidential urine sample within one hour. In addition to urine providing a less contemporaneous toxicological perspective than blood, the road-traffic urine procedure is frequently subject to legal challenges.^27^ Therefore, the preference for most law-enforcement agencies in the UK is to collect blood where possible, suggesting that capillary blood extraction and micro-sampling analysis could offer a valuable alternative to current practice.

Previous research investigating micro-sample testing of blood alcohol at lower sample volumes suggested this is a viable technique.^23–25^ Wilkinson et al. described a headspace GC-FID method for ethanol analysis utilising 20–50 μL blood samples with a reported average precision of 4.6% and a concentration range of 0.003–1.2 mg/mL.^24^ A study by Vance et al. investigating GC-FID analysis of blood alcohol utilised a 50 μL sample volume with a 1 mL internal standard volume and an acceptance criterion that the duplicate ethanol results must be within 5% or 5 mg/100 mL.^25^ Our results detail the CV and intra-sample variation of 10 μL volumes of venous and capillary blood. The CV of 10 μL venous blood samples was 2.54%, and the CV of 10 μL capillary blood samples was 3.21%. Neither of the methods employed by Vance et al. and Wilkinson et al. compare the accuracy or viability of micro-sampling with traditional sample testing. However, they do demonstrate the validity of the technique and support the results of this experimentation.^24,25^ An alternative method for BAC analysis employing ^1^H NMR was developed by Zailer and Diehl, where they utilised 20 μL blood for the analysis of alcohol with their analysis method, examining concentrations within the range 0–3 g/L.^23^ While this method displayed an impressive sensitivity on a relatively small sample, the use of ^1^H NMR for volume toxicology analysis is not cost-effective or commercially viable.

In addition to investigating the viability of reduced sample volumes in the analysis of BAC, we also examined the differences between venous and capillary blood one hour after cessation of drinking. The data show a statistically significant difference (*p* ≤ 0.05) between the BAC of venous and capillary blood samples. The measured alcohol content of almost all capillary blood samples was lower than that of the corresponding venous blood samples. This applies for individual aliquots as well as the full population data (Table 6). An average venous versus capillary difference of approximately 3.42 ± 1.96 mg/100 mL at 100 μL and approximately 4.29 ± 2.29 mg/100 mL at 10 μL was observed. Previous work carried out by Jones et al. examined the differences in alcohol content of venous blood compared to capillary blood, and they focused on how different sampling times and the source of the blood (capillary or venous) influenced BAC.^15^ This study suggested that whilst in the absorption phase, capillary blood alcohol was higher than the corresponding venous BAC. However, once the post-absorptive phase was reached (after approximately 90 minutes), venous BAC was higher. The average capillary–venous difference was 5.8 ± 3.4 mg/100 mL, and this did not appear to change significantly for the remainder of the 390-minute experimentation period. The post-absorptive capillary and venous blood-alcohol variation described by Jones et al. is comparable to the results in this study. This suggests that there is a reliable correlation between the two blood sources. Jones et al. compared alcohol levels in venous and capillary blood one hour after the start of alcohol consumption, while this study looked at differences one hour after cessation of drinking, as one hour is an approximate time of the completion of the alcohol absorption phase.^15,28,29^ Furthermore, this sampling procedure better reflects the process of collecting samples for road-traffic toxicology analysis where sample collection only occurs after at least one hour has elapsed since the suspect last consumed alcohol.

**Table 6.** Wilcoxon matched-pair signed rank test for all subject data at each sample subset.

Sample subset	Number of samples	Mean difference ± *SD* (mg/100 mL)	95% CI for mean difference	Wilcoxon signed rank test *p*-value
Comparison of BAC of 100 µL and 10 µL aliquots of venous whole blood	39	0.41 ± 1.94	–0.22 to 1.04	0.209
Comparison of BAC of 100 µL and 10 µL aliquots of capillary whole blood	38	1.21 ± 1.88	0.59 to 1.83	0.001
Comparison of BAC of 100 µL and 100 µL aliquots of venous and capillary whole blood	39	3.38 ± 1.99	2.74 to 4.03	<0.001
Comparison of BAC of 10 µL and 10 µL aliquots of venous and capillary whole blood	38	4.13 ± 2.42	3.34 to 4.93	<0.001

Non-parametric statistical analysis was used to analyse the statistical significance of BAC from differing sample sites and different blood volumes analysed. All sample subsets, with exception of venous 100 µL blood compared to venous 10 µL blood, were found to be significantly different.

CI: confidence interval.

A limitation of this study was the use of only one time point for sampling. Utilising more time points, perhaps one before the hour, at 30 minutes post consumption and a third time point at 90 minutes after consumption would allow for a more comprehensive analysis of the venous and capillary profile and further corroborate the work of Jones et al. regarding the venous and capillary difference during and after the absorption phase. A further limitation of this study was the lack of high BAC readings to compare the difference in venous versus capillary BAC at higher ranges in relation to lower levels. The addition of volunteers with BACs >80 mg/100 mL would provide a better understanding of the relationship of BAC to the venous and capillary blood alcohol, and this will be expanded upon in future research. Another limitation of this study and the subject of further research is the lack of demographic variation of volunteers in this study. Moreover, a higher number of analyses with a larger number of participants would help to verify this proof of concept. Increasing the sample size of the participants would further establish and confirm the statistical uncertainty and accuracy of utilising capillary blood for BAC analysis.

A potential difficulty of using capillary blood is extracting sufficient quantities for traditional analysis techniques, which typically use 0.1–1 mL of sample in duplicate. Capillary blood, whilst easier to obtain in small volumes, becomes more difficult when larger volumes are needed. The difficulty is, however, mitigated by utilising a 10 μL sample volume, which reduces the volume of sample required for an analysis whilst maintaining a comparable sensitivity to higher sample volume analyses.

The varied nature of the differences between capillary and venous values in this experimentation could be attributed to an unstandardised specification on fasting state and food intake prior to alcohol ingestion, as in this study, volunteers were not required to fast before the experiment began. This could have played a role, as volunteers would have a varied speed of gastric emptying, with some volunteers having eaten hours before the experimentation and some having not eaten for an extended period of time by comparison. Different meal compositions may also have played a part in this, with higher carbohydrate or fatty foods being a contributor to a slower gastric emptying rate. This could alter the time taken for the post-absorptive phase of ethanol to be reached.^28,29^ This was an intentional feature of the study in order to provide a cross-sectional analysis of a simulated real-life scenario, whereby suspects calorific and dietary intake will vary on a case-by-case basis, thus increasing inter-subject variability. This has the consequence of raising pre-analytical variability. A further cause of variation is that volunteers also consumed different volumes of different alcoholic beverages, with some consuming just one pint and others consuming a second, thus giving a higher variability of BAC values.

In conclusion, it has been found that a sample size of 10 μL is a viable method of sampling for both venous and capillary blood samples. A plausible benefit of using micro-samples for blood-alcohol analysis is that there is the potential for a rapid, simpler and more efficient collection procedure from detainees in custody. There is also no specification in UK law on where blood samples should be collected from on a drink-driving suspect.^30,31^ Therefore, capillary blood could be lawfully collected and analysed for BAC. Our results indicate that there is on average a 3.85 mg/100 mL increase in alcohol concentration for venous samples compared to capillary blood samples one hour after cessation of drinking. Therefore, while faster collection times using capillary blood may be of benefit in detecting blood alcohol prior to elimination, they are likely to provide some underestimation of alcohol content when compared to venous blood. Regardless, this technique may still be useful in cases of poor venous access or inability of the patient to provide a venous sample.
